# Characteristics of two questionnaires used to assess interprofessional learning: psychometrics and expert panel evaluations

**DOI:** 10.1186/s12909-018-1153-y

**Published:** 2018-03-20

**Authors:** Samuel Edelbring, Madeleine Abrandt Dahlgren, Desiree Wiegleb Edström

**Affiliations:** 1 Department of Medicine and Health Sciences, Linköping University, 581 83 Linköping, Sweden; 2 Department of Learning, Informatics, Management and Ethics, Karolinska Institutet, 171 77 Stockholm, Sweden; 3 Dermatology Unit, Department of Medicine Solna, Karolinska Institutet, Stockholm, 171 77 Stockholm, Sweden; 4 Department of Dermatology, Karolinska University Hospital, Stockholm, 171 76 Stockholm, Sweden

**Keywords:** Interprofessional learning, Competencies, Assessment, Questionnaires, Validation

## Abstract

**Background:**

Interprofessional learning activities are included in many curricula but are difficult to assess. For languages that are not widely spoken such as Swedish, few validated questionnaires exist that relate to interprofessional outcomes. Therefore, the aim was to examine two such questionnaires in relation to interprofessional competence domains.

**Methods:**

Psychometric characteristics, such as homogeneity of items and internal consistency, were assessed for the Swedish versions of the *Jefferson Scale of Attitudes Towards Physician-Nurse Collaboration* (JSAPNC) and the *Readiness for Interprofessional Learning Scale* (RIPLS). The questionnaires were distributed directly following IPL activities. Mokken scale analysis based on Loevinger’s coefficient for homogeneity and Cronbach’s alpha were used to evaluate the scales. Two expert panels performed a qualitative analysis of items in relation to four internationally defined interprofessional competences.

**Results:**

In total, 88 and 84 responded to the JSAPNC and RIPLS questionnaires, respectively. Estimates of homogeneity were low for both the JSAPNC (H = 0.16) and the RIPLS (H = 0.21). Reliabilities were weak (0.62 and 0.66, respectively) for the total scales. The expert panels categorised 68% of items into similar competence domains. However, their discussion revealed ambiguous wordings and imbalances in the two questionnaires in relation to domains.

**Conclusion:**

Interprofessional competence domains are defined but few validated tools exist to assess them. Examined tools relating to interprofessional learning in Swedish do not qualify for assessing overarching IPL outcomes, and summed scores from these tools should be used with caution.

## Background

Interprofessional collaboration has been regarded as necessary for addressing the complexity of patients’ needs and is considered a crucial element of contemporary healthcare [1]. Educational efforts are thus needed to prepare learners for collaboration with other professionals and must be adequately assessed to ensure quality and proper development. Following the increased integration of interprofessional learning activities into curricula, the need for tools for assessing overarching interprofessional competences across health professions has been voiced [2].

Outcomes of interprofessional learning (IPL) can be expressed in various ways with respect to different professional areas as well as different competences. Freeth et al. [3] identified the following hierarchical levels of possible outcomes of interprofessional education: reaction, attitudes and perceptions, knowledge/skills, behavioural change, change in organisational practice and benefits to patients. Studies in undergraduate IPL often report outcomes in the first two levels, and the majority of IPL studies (70%) typically measure these outcomes using questionnaires [3]. Questionnaires are convenient to distribute and straightforward to analyse and report on, which could explain their frequent use. The underlying assumption is that questionnaires cover important aspects of IPL, while their psychometric properties ensure the valid interpretation of their results. However, evaluations of the validity and reliability of several types of questionnaires used to assess IPL in fact demonstrate that their psychometric integrity is limited and suffers from ceiling effects, reducing the possibility of detecting changes in IPL outcomes [4].

IPL is most effective when embedded within an overarching programme, of which assessment forms an integral part [5]. Such a programme should aim towards providing students with core competences for interprofessional practice. Over the years, core competencies have been identified in interprofessional competence frameworks developed in different countries. The Interprofessional Education Collaborative’s (IPEC) outlined interprofessional collaborative practice is in four domains: *values/ethics for interprofessional practice*, *roles/responsibilities*, *interprofessional communication* and *teams and teamwork* [6, 7]. The competences described in this framework thus portray the capabilities that characterise a health professional skilled in interprofessional collaboration. The IPEC conceptual framework is widely accepted and has also inspired the development of several IPL curricula for students in, for example, Sweden [8] and the US state of Nebraska [9]. Other competency frameworks exist, broadly describing IPL aims in a similar manner [2, 10]. Overarching intended outcomes of IPL are thus identified in broad agreement. Establishing which methods to use for assessing these outcomes in relation to accepted domains is, however, a challenging task.

Reviews of IPL research demonstrate large variations of research methodologies for assessing IPL outcomes [11, 12]. Such variation is expected because different approaches to IPL require different methods for its evaluation [13]. However, a lack of widely accepted instruments has also been identified [14]; many instruments are developed and used only once. In fact, most existing instruments lack rigorous assessment of their psychometric properties [15]. Internationally used, validated and reliable IPL measures could help to improve IPL activities and thus contribute to improved healthcare [15, 16]. A reasonable expectation would be that questionnaires would cover parts of some of the aforementioned domains and thus contribute to important parts of a programmatic IPL curriculum as well. Anderson et al. [5] proposed the role of questionnaires as establishing a baseline for regular assessment of change.

Most validity and reliability assessments are carried out in English-speaking countries. Evaluations of how these scales translate into other languages sometimes remain unexplored. A literature search in PubMed and reviews on IPL surveys [14, 17] yielded two evaluated questionnaires for undergraduate IPL that are available in Swedish: the Readiness for Interprofessional Learning Scale (RIPLS) [18] and the Jefferson Scale of Attitudes Towards Physician-Nurse Collaboration (JSAPNC) [19]. Both of these questionnaires have been extensively used internationally and have, in general, been identified as useful tools for assessing IPL e.g. [17, 20–22]. The RIPLS is the most frequently used IPL tool and addresses interprofessional learning and attitudes towards collaboration between health professionals in general [16]. Weaknesses have, however, been reported regarding, e.g., its unstable scale structure and ability to detect differences between pre- and post-IPL activities [23, 24]. Meanwhile, the JSAPNC has undergone much assessment but no substantial criticism has been voiced regarding its use. This scale addresses attitudes towards collaboration between nurses and physicians. The JSAPNC has previously been used in the Swedish context for, e.g., comparing Swedish medical students from two universities regarding their attitudes towards physician-nurse collaboration [25], while the RIPLS has been used more frequently to assess the benefits of IPL [20, 26, 27]. Results from these scales are often interpreted from an overall sum score. It is thus important to scrutinise whether the items contributing to the sum score represent the expected phenomena, i.e., the homogeneity of the scale. Weak homogeneity presents a risk when interpreting sum score values because, in such a case, items contributing to the sum score could represent different underlying phenomena.

The aim of this study was to improve knowledge about using questionnaires to assess IPL in Sweden. Two research questions were formulated to fulfil this aim: (1) What are the psychometric properties of Swedish versions of internationally validated IPL questionnaires? and (2) How do these questionnaires relate to interprofessional competence domains?

## Methods

### Contexts for the two study settings

Student response data were collected at two health profession schools in Sweden, each using one questionnaire. In Sweden, IPL was pioneered by the Faculty of Medicine and Health Sciences at Linköping University but is now promoted by all health profession schools and programmes. A national examination requirement states that health profession students should be able to show teamwork skills and be able to collaborate with other professionals. At one of the two study sites, Karolinska Institutet in Stockholm, IPL occurs differently in the various study programmes, generally focusing on teamwork with some progression consecutively throughout the study programmes. A two-week placement on a student-led interprofessional training ward is a mandatory part of the later years of four programmes. The other study site, the Faculty of Medicine and Health Sciences at Linköping University, adopts an outspoken IPL profile, and integrates IPL in three phases involving students from a broad range of health professions [28]. Students from six study programmes participate in these activities, of which the last one consists of a student-led interprofessional training ward.

The JSAPNC was introduced in 2013 in order to assess interprofessional learning in a single IPL activity for 2nd year nursing and 3rd year medical students. The JSAPNC was chosen in this setting because of its international widespread use and psychometric merit [29], but had not previously been utilized in this setting. The RIPLS had been previously used in this school in conjunction with the student-led interprofessional ward.

### Psychometric evaluations

A psychometric evaluation was performed on student responses using Mokken scale analysis, which uses Loevinger’s homogeneity coefficient H to measure unidimensionality, i.e., the degree to which a group of items measures the same underlying construct [30]. Mokken analysis falls into nonparametric item response theory (IRT) and was developed for questionnaire item analysis. It sets less strict assumptions on data compared to parametric IRT models and factor analyses, making it suitable for complex social science constructs, such as attitudes related to IPL [31]. The rule of thumb regarding the homogeneity coefficient H is a threshold value of 0.3. An estimate between 0.3 and 0.4 for a group of items is considered to have weak unidimensionality, while H > 0.4 and H > 0.5 are characterised as having medium and strong dimensionality, respectively. The reliability was measured using Cronbach’s alpha. The rule of thumb measure for a reliable scale is 0.7 [32].

Responses from the JSAPNC were gathered from nursing and medical students (semesters four and six, respectively) at Karolinska Institutet, Stockholm. The data were gathered directly after an IPL activity and data from three consecutive semesters during the period 2013–2015 was pooled into one dataset. Two minor adjustments were made for readability reasons: “physician” instead of “doctor” and “discussed” instead of “clarified”, to items 8 and 13, respectively.

Responses from the RIPLS were gathered in 2011 from nursing and medical students in their final year after a full day IPL simulation activity at the Faculty of Medicine and Health at Linköping University.

### Questionnaire correspondence to interprofessional competence domains

In order to balance evaluation data based on student responses to the IPL-related questionnaires, judgments from IPL teachers was sought via two expert panels. Experienced IPL educators and researchers from the faculty with an IPL profile were recruited to assess the items in relation to IPL competence domains. Invitations to voluntarily participate were sent by email to teachers involved in interprofessional education. In order to increase variability and improve the availability of participants, two sessions were arranged. In total, 14 experts (11 at the first session and three at the second) with professional backgrounds as physicians, physical therapists, occupational therapists and nurses participated. The two questionnaires and the IPEC domains (*values/ethics for interprofessional practice*, *roles/responsibilities*, *interprofessional communication* and *teams and teamwork*) were presented, after which questionnaire items were distributed. One copy of each questionnaire item – in total, 34 items (15 and 19 for the JSAPNC and RIPLS, respectively) – was evenly distributed among the participants. The item wordings were read aloud by each participant in turn, and each item was discussed in relation to the four competence domains. Following a consensus discussion in the group, the item was categorised to one of the IPEC domains by the person reading the item. Each domain was represented by a box labelled with the domain name along with a general competency statement and criteria explaining the domains as formulated by IPEC [6]. Items categorised into the same category by the two panels were synthesised into a common table. One of the authors (SE) observed and took notes on the discussion and comments, summarizing characteristics of the discussions.

## Results

### Psychometric analyses

#### JSAPNC

Eighty-eight participants, comprising 37 nursing and 51 medical students, answered the JSAPNC (95% of the present participants at the IPL activity). Mokken scalability for the complete scale was very low (H = 0.16), indicating a multidimensional structure in the items. Of the four subscales, three displayed unidimensionality above the threshold level, ranging from weak to strong scalability (Table 1). The reliability was weak, ranging from 0.40 to 0.63 for the subscales and 0.62 for the overall scale.

**Table 1:** Reliability and homogeneity for JSAPNC and its subscales

Subscales	Shared Educational Experiences and Professional Collaboration	Caring as Opposed to Curing	Nurse’s Autonomy	Physician’s Authority	All Items
Items	1, 3, 6, 9, 12, 14, 15	2, 4, 7	5, 11, 13	8^a^, 10^a^	1–15
Cronbach’s alpha	0.46	0.44	0.40	0.63	0.62
Homogeneity (Coef. *H*)	0.18	0.44	0.32	0.54	0.16

(^a^Scores reversed as recommended because of negative item wordings.) *N* = 88

#### RIPLS

Eighty-four participants, comprising 20 nursing and 64 medical students, answered the RIPLS (all the present participants at the IPL activity). Ten questionnaires were discarded from the analysis because of missing values, leaving *n* = 74 complete questionnaires. Mokken scalability for the complete scale was very low (H = 0.21). Of the three subscales, two displayed unidimensionality above the threshold level, with weak unidimensionality (Table 2). The reliability ranged from very low (0.36) to high (0.79) for the subscales and 0.66 for the overall scale.

**Table 2:** Reliability and homogeneity for RIPLS and its subscales

Subscales	Teamwork and collaboration	Professional identity	Roles and responsibilities	All items
Items	1, 2, 3, 4, 5, 6, 7, 8, 9	10^a^, 11^a^, 12^a^, 13, 14, 15, 16	17, 18, 19	1–19
Cronbach’s alpha	0.79	0.75	0.36	0.66
Homogeneity (Coef. *H*)	0.38	0.34	0.19	0.21

(^a^Scores reversed as recommended because of negative item wordings.) *N* = 84

### Expert panels’ assignment of items to core competences

The expert panels found that both the JSAPNC and RIPLS to some extent matched the four IPEC core competences of interprofessional collaborative practice. However, variation was found between item assignments between the two sessions (Table 3). Selecting categories was sometimes challenging to perform and caused lively discussion on item wordings and interpretations in relation to the four domains.

**Table 3:** Expert panel’s separate and joined categorisations of items from JSAPNC (J-item no. in table) and RIPLS (R-item no. in table) into core interprofessional competencies

Values/ethics for interprofessional practice	Roles/responsibilities	Interprofessional communication	Teams and teamwork
First expert panel’s categorisation			
J13	J1	J15	J9
R6	J2	R3	J11
R7	J3	R5	J14
R10	J4	R13	R1
R19	J5		R2
	J6		R4
	J7		R8
	J8		R11
	J10		R12
	J12		R14
	R9		R15
	R18		R16
			R17
Second expert panel’s categorisation			
J8	J1	J14	J3
J11	J2	J15	J9
R4	J4	R3	R1
R6	J5	R5	R2
R7	J6	R9	R8
R10	J7	R13	R14
R11	J10	R15	R16
R12	J12		
R19	J13		
	R17		
	R18		
Common JSAPNC and RIPLS items in relation to core interprofessional competencies from the two IPL expert panel sessions			
R6	J1	J15	J9
R7	J2	R3	R1
R10	J4	R5	R2
R19	J5	R13	R8
	J6		R14
	J7		R16
	J10		
	J12		
	R18		

Twenty-three out of 34 items were assigned to the same domains by the two panels. Of these common categorisations, most items concerned *roles/responsibilities* (*n* = 9), followed by *teams and teamwork* (*n* = 6); the two other categories were assigned four items each (Table 3, common items). All except one of the *roles/responsibilities* items were assigned from the JSAPNC questionnaire. However, no JSAPNC item was assigned to *values/ethics* and only one JSAPNC item was assigned to each of the *interprofessional communication* and *teams and teamwork* domains, which were instead dominated by RIPLS items.

In the first session, more than half the RIPLS items were assigned to *teams and teamwork*. Three items from the RIPLS subscale *teamwork and collaboration* were assigned to the *teams and teamwork* competence domain in both sessions, and two items from the JSAPNC subscale *shares education and collaboration* were found in *roles/responsibilities.*

The items were generally found to be formulated in a socially desirable way: ‘It would be impossible for my students *not* to endorse these items’ as one participant commented. Comments were also made about the focus on physician and nurse professions, in which representation of other professions was requested. The nurse-physician relationship reflected in many items was described as ‘an underdog perspective that needs to change’.

The patient perspective was identified as lacking. Panel participants also asked about the theoretical foundations behind the items. Some of the item wordings were regarded as ambiguous, with the expert group reacting negatively to the wording of some items. The wordings were found to be awkward in relation to their own educational context, and some wordings implied a stronger professional hierarchy than the participants knew from their own Swedish practice. Some expressed, for example: ‘One can tell that this item originates in another system’ and ‘I would never allow a student of mine to use such questions in their own survey’.

## Discussion

Data from student responses and IPL educators provided an assessment of scale dimensionalities and the relationships between items in the two selected questionnaires and the overarching interprofessional competence domains. The most striking result was the low homogeneity among items in both questionnaires when considered as whole scales; in addition, neither questionnaire covered all four important interprofessional competence domains.

Neither the JSAPNC nor the RIPLS displayed levels of homogeneity above the threshold for weak scales (coefficient H > 0.3). Internal consistencies were also low in the data from both questionnaires, slightly below the rule of thumb value for reliability (alpha 0.7). Comparisons with other Swedish findings are difficult to perform because of a lack of homogeneity and reliability reports. Only one previous report was found with total RIPLS reliability in a Swedish setting, which had a Cronbach’s alpha of 0.62 [33]. In other Swedish studies, only subscale values were reported e.g. [20], demonstrating estimates above 0.7 for only one subscale, *teamwork and collaboration.* These low estimates and the lack of reported total reliability indicates that reliability in Swedish settings is generally lower than in international settings where total estimates of, e.g., 0.90 [18] and 0.85 [34] have been reported. No previous Swedish reliability estimates for the JSAPNC were found, but a study in American, Israeli, Italian and Mexican contexts observed Cronbach’s alpha estimates ranging from 0.70 to 0.86 [35]. Reliability is necessary but not sufficient for unidimensionality in a scale [36]. The JSAPNC and RIPLS both suffered from weak reliabilities and low dimensionality estimates in the Swedish samples, thereby undermining expectations of unidimensionality in this national setting. The different national settings, between where the questionnaires originated (the UK and the US) and Sweden, may influence content validity. This may be of particular concern for the JSAPNC, in which the relationship between physicians and nurses dominates the content.

The four IPEC domains comprised a structured approach to examining these two questionnaires. The two expert panels’ discussions revealed ambiguous wordings, making categorisations of items difficult in some cases. Several items sparked animated discussions, and the two panels related some items to different domains. One explanation for this result is that an item’s content could be related to more than one domain. For example, the wording of item 3 on the JSAPNC could be related to either domain 4 (*teamwork*) or domain 2 (*understanding of roles*): *‘During their education, medical and nursing students should be involved in teamwork in order to understand their respective roles’.* Nevertheless, each domain was found to have at least four items in common by the two panels. An American interprofessional group of educators also mentioned experiencing difficulties mapping RIPLS items to IPEC domains [9].

The domain *roles/responsibilities* represented the largest group of common items. Notably, all except one item in this group were JSAPNC items. Of these, items from all JSAPNC subscales were represented. Thus, as a whole, the JSAPNC could be said to relate very much to *roles/responsibilities* while displaying weak relationships to the other three domains. This may be because the JSAPNC relates specifically to collaboration between nurses and physicians, which makes it natural to relate wordings to *roles and responsibilities*. Only one RIPLS item (17) mentions specific professions. Responses on the nurse-physician relationship are related to respondents’ own cultural contexts. The American origin of JSAPNC is described as having a complementary model of physician-nurse relationship [35]. However, differences in hierarchical levels between these professions are known to vary, and there are reasons to believe that the Swedish relationship model is different, which implies consequences for interpretations of this subscale scores.

The imbalance of JSAPNC/RIPLS item representation found in the *roles/responsibilities* domain is opposite to that of the other three domains, which were dominated by RIPLS items. Consequently, the RIPLS scale could be said to relate to most of the IPEC domains. The lack of RIPLS items related to *roles and responsibilities* is remarkable given the importance of this domain for IPL [37]. The RIPLS does have a subscale called *roles and responsibilities*, the items of which would be expected to relate to this domain. This subscale received weak psychometric support in earlier studies, which could explain why it did not represent itself clearly in the panels’ assessment. Two of these three subscale items were related to the *roles/responsibilities* domain by the second panel session, but only one was assigned this relationship by both sessions. Mahler et al. [23] summarised reports of reliabilities below 0.43 and mentioned that several researchers omitted the subscale altogether in total scores. Given its importance, it is problematic that this dimension of IPL does not function well in the RIPLS. Understanding one’s role as a health professional and how it contributes to patient care is part of most intended IPL outcomes and needs to be addressed in health profession curricula [38, 39].

Taken together, our data show that the two questionnaires do not meet rigorous demands in assessing overarching IPL aims. While they are relevant in addressing attitudes towards interprofessional learning (i.e., the RIPLS) and profession-specific attitudes towards collaboration (i.e., the JSAPNC), they demonstrated weak psychometric properties in our samples and did not fully address overarching IPL aims.

The two questionnaires both address attitudes and perceptions, i.e., what could be categorised under lower hierarchical levels of IPL outcomes as described by Freeth et al. [3]. However, we argue that attitudes are related to competence and readiness for action and, therefore, merit assessment insofar as students’ readiness for collaborative practice is concerned. The IPEC domains present strong links to professional competence and behaviour, and may serve as a basis for the further development of assessment tools. The RIPLS and JSAPNC relate to IPEC domains but are not explicitly developed to target these. In order to connect more strongly to overarching IPL aims, Swedish existing tools need to be adapted or new ones developed. Two noteworthy American efforts have been made to develop tools relating to interprofessional competencies as defined by IPEC: Dow et al.’s [40] work, in which 42 items were mapped directly to the four domains, further refined and shortened [41], and Beck Dallaghan et al.’s tool, in which IPEC domains were used to develop a four-factor model comprising 19 items [9]. These tools could be promising candidates for the development of Swedish scales because of their strong connection to overarching IPL objectives. The validity of the four selected target domains is supported by global consensus [6]. However, even though these core IPL aspects seem to be stable, an IPL curriculum development group using this framework identified the need for a fifth domain reflecting *learning* [8]. A recent global panel of experts also identified the need for *coordination and collaborative decision-making* and *reflexivity* categories, in addition to domains similar to those in the IPEC [42]. Consequently, these categories should be taken into consideration in future revisions to or the development of new scales to measure IPL.

### Methodological considerations

Given that the interprofessional competencies and IPL include collaborations between many professions, it is important to consider that psychometric data originates from only from two professions, thereby limiting the conclusions that can be drawn from these. Furthermore, the psychometric evaluations were made in single settings with modest sample sizes. For more far-reaching conclusions, additional samples with larger numbers of students should be included. The expert panels’ evaluation was based on both individual and group perceptions of relationships between single items and the four IPEC domains. Consequently, it was not an evaluation of how well items assessed the content of the domains, and individual rater variations in perception influenced the outcomes. The consensus group method also risks being biased by dominant individuals and perspectives. The delegation of the final decision on each item categorisation was distributed equally amongst participants in order to balance this potential bias.

A conflict between expectations of the two evaluation approaches is possible. On the one hand, psychometric evaluation assumes unidimensional scales measuring a single phenomenon with high precision. On the other hand, the IPEC framework used in the expert panels’ evaluation assumes coverage of broad domains. Considering the composite nature of interprofessional competences and the mixed characteristics of respondents, an expectation of unidimensionality in IPL questionnaires is likely futile.

### Concluding remarks and implications

A hypothetical fully unidimensional scale would likely be so reductionist and stereotypical in terms of wording that it would lose relevance. Considering the intended learning outcomes of IPL, a single unidimensional scale is not even desirable. The resolution to this dilemma could be to first consider the multidimensional characteristics underlying these phenomena and subsequently place more weight on subscale scores while refraining from the use of total sum scores from the whole questionnaire. This practice is possible when subscales display adequate scalabilities and relate to meaningful IPL aspects.

In these two Swedish IPL settings, neither of the questionnaires’ response data displayed strong psychometric properties. Notably, the scalability of each questionnaire was low meaning that the summed total scores for each student do not represent one underlying phenomenon in the respective context. Summed scores for these questionnaires in similar settings should thus be interpreted with caution because the items comprising the whole set could relate to different, possibly unrelated, aspects of IPL. Some subscales displayed adequate scalabilities, but these scales do not replicate well in educators’ interpretations of established interprofessional competencies.

## Conclusions

There is consensus about the aims of interprofessional learning in terms of interprofessional competences. However, few validated tools exist to assess such competences. A closer look at IPL tools available in Swedish show that they are insufficient for evaluating interprofessional competences. The use of total summed scores of these questionnaires is discouraged since items comprising the whole set appear to measure different IPL aspects.

## Abbreviations

IPEC: The Interprofessional Education Collaborative
IPL: Interprofessional learning
JSAPNC: Jefferson Scale of Attitudes Towards Physician-Nurse Collaboration
RIPLS: Readiness for Interprofessional Learning Scale
